# The diverse virulence potential of atypical enteropathogenic *Escherichia coli* isolated from diarrhea: the emergence of a hybrid pathotype?

**DOI:** 10.3389/fmicb.2025.1599350

**Published:** 2025-06-06

**Authors:** Ana C. M. Santos, Roberta S. Silva, Mônica A. M. Vieira, Cristina V. Niero, Matheus S. F. Ribeiro, Beatriz E. C. Guth, Tânia A. T. Gomes, Rosa M. Silva

**Affiliations:** ^1^Laboratório de Enterobactérias, Departamento de Microbiologia, Imunologia e Parasitologia, Escola Paulista de Medicina, Universidade Federal de São Paulo, São Paulo, Brazil; ^2^Laboratório Experimental de Patogenicidade de Enterobactérias, Departamento de Microbiologia, Imunologia e Parasitologia, Escola Paulista de Medicina, Universidade Federal de São Paulo, São Paulo, Brazil; ^3^Laboratório de Biologia Molecular de Micobactérias, Departamento de Microbiologia, Imunologia e Parasitologia, Escola Paulista de Medicina, Universidade Federal de São Paulo, São Paulo, Brazil

**Keywords:** ExPEC, UPEC, aEPEC, children diarrhea, *Escherichia coli*, hybrid pathogenic, hetero-pathogenic

## Abstract

Despite being in the era of advanced technology, the world still suffers from old infectious diseases, both intestinal and extraintestinal, where *Escherichia coli* plays a major role as the etiological agent. Atypical enteropathogenic *E. coli* (aEPEC) is one of six intestinal pathogenic *E. coli* pathotypes and one of the major agents causing diarrhea in low- and middle-income countries like Brazil. In this work, we have investigated to what extent a collection of aEPEC isolated from the intestinal tract of children has incorporated virulence traits involved in the development of extraintestinal infections. The phylogenetic origin and the presence of extraintestinal pathogenic *E. coli* (ExPEC) -related Pathogenicity Islands (PAIs) were evaluated by PCR for a collection of 111 aEPEC isolated from stool. Additionally, they were screened by PCR for the presence of specific ExPEC virulence factors. Phenotypically evaluated for bacteriocin and hemolysin production and assessed for serum resistance. Finally, four strains were sequenced and had their genome characterized. Most of the strains originated from phylogroup B1 (48.6%) and A (36.3%), followed by groups B2 (13.5%) and E (8.1%). About half of the aEPEC strains presented markers for pathogenicity islands originally described in uropathogenic *E. coli* (UPEC), PAI IV536 being the most prevalent. Many aEPEC strains presented the virulence genetic markers that are the hallmark of ExPEC. Besides, many strains produced bacteriocins and hemolysins and survived in human serum. Five strains fulfilled the molecular criteria to be classified as ExPEC and one as UPEC, highlighting the existence of hybrid genotypes among aEPEC strains. Three non-phylogenetic-related hybrid strains were chosen for further experiments. These strains were lethal in the *Galleria mellonella* model for ExPEC virulence, and the comparative analysis of their genomes revealed they belong to different EPEC/ EHEC global clonal groups. Overall, this study reports the presence of many attributes of virulence of ExPEC in a comprehensive collection of aEPEC strains. The data presented here indicate the existence of genotypic hybrid aEPEC/ExPEC and aEPEC/UPEC pathogens, suggesting that they can express both intestinal and extraintestinal virulence determinants in humans. Therefore, the consequences of their colonization and infection are more concerning and potentially life-threatening.

## 1 Introduction

Diarrhea is still the second leading cause of death in children under 5 years old (WHO, 2017). According to the World Health Organization (WHO), in the past, diarrhea-related deaths were due to severe dehydration and fluid loss; however, currently, septic bacterial infections are pointed out to account for an increasing proportion of all diarrhea-associated deaths (WHO, 2017).

The mortality and morbidity rates of Gram-negative septic infections are high, and *E. coli* is a leading cause of bloodstream infections worldwide. Some virulence factors (VFs) present in mobile genetic elements can be incorporated into diverse genomic backgrounds, enabling pathogens to cause different diseases. In this sense, the emergence of hetero-pathogenic or hybrid pathogenic *E. coli* emphasizes the possibility of some strains causing diarrhea and systemic infections in the same host (Mariani-Kurkdjian et al., 2014; Kessler et al., 2015; Mandomando et al., 2020; Santos et al., 2020a). Therefore, a portion of the septic infections related to diarrhea caused by *E. coli* can be associated with strains that have caused diarrhea and then, taking advantage of the intestinal barrier impairments, translocate and reach extraintestinal sites, causing bloodstream infection in the host. This pathogen can be either an Intestinal Pathogenic *E. coli* (IPEC) that received VFs from Extraintestinal Pathogenic *E. coli* (ExPEC) or vice versa.

Enteropathogenic *Escherichia coli* (EPEC) is one of the six IPEC pathotypes and is a leading cause of diarrhea in low- to middle-income countries like Brazil (Afset et al., 2004; Aranda et al., 2007; Gomes et al., 2016).

The EPEC pathotype causes diarrhea due to the presence of a pathogenicity island named Locus of enterocyte effacement (LEE) (Denamur et al., 2021). The LEE contains several genes that encode virulence factors (VFs), which work in coordination with additional genes in the genome to induce bacterial intimate adherence to enterocytes, the cell’s actin remodeling, and the rise of pedestal-like structures below the adhered bacteria, culminating in the microvilli elimination in the region surrounding the bacterial attachment (Trabulsi et al., 2002; Hernandes et al., 2009; Gomes et al., 2016). The EPEC pathotype can be subdivided into two subgroups based on the expression of an adhesin responsible for facilitating adherence onto the enterocyte, named bundle-forming pilus (BFP), which is present in strains classified as typical EPEC (tEPEC) and absent in those classified as atypical EPEC (aEPEC) (Trabulsi et al., 2002; Kaper et al., 2004; Gomes et al., 2016). In Brazil, aEPEC strains represent about 50% of the diarrhea caused by *E. coli* (Ori et al., 2019). Moreover, aEPEC pathotype genome is recognized to be permissive to the incorporation of DNA by horizontal gene transfer (HGT) events (Afset et al., 2008), which leads to a very heterogeneous array of virulence genetic markers displayed by the aEPEC strains. Recent reports have highlighted aEPEC isolates from extraintestinal infections, including bloodstream infections that may or may not be associated with diarrhea (Bratoeva et al., 1994; Kessler et al., 2015; Valiatti et al., 2020; Nascimento et al., 2021, 2022).

In this work, we evaluated diverse aspects related to the ExPEC pathotype in an aEPEC collection isolated from stools in diverse epidemiological studies to search for the presence of ExPEC-recognized virulence traits that could reveal the existence of hybrid intestinal pathogenic strains with the potential to cause both intestinal and extraintestinal infections.

## 2 Materials and methods

### 2.1 Bacterial strains

In this study, 111 previously published aEPEC strains isolated during epidemiological studies conducted in Brazil were revisited (Vieira et al., 2001, 2010; Gomes et al., 2004). Seventy-eight strains (70.3%) were isolated from diarrhea cases and 33 (29.7%) from asymptomatic individuals. The EPEC pathotype was determined by the presence of the *eae* (intimin) gene and the absence of all the other genes related to any other IPEC pathotype, while the atypical subclassification was determined by the lack of the *bfpA* (BFP) gene (Vieira et al., 2001, 2010; Gomes et al., 2004).

### 2.2 Bacteriocin production

The production of bacteriocins was assessed as described earlier (dos Santos et al., 2017). Briefly: strains were grown on three different solid culture media: Nutrient agar (NA), Tryptone yeast extract (TYE) (Difco Laboratories, Sparks-MD), and TYE plus 1% trypsin (Gibco^®^) (TYE-T). The indicator strain *E. coli* C600, grown overnight (ON) in Tryptic Soy broth (TSB) (Difco), was diluted 1:100 in sterile saline solution (0.85% NaCl) and spread on the surface of the TYE, NA, and TYE-T media plates to obtain a bacterial lawn. Three microliters of ON TSB cultures of aEPEC strains were seeded over the indicator lawn in equidistant spots. *E. coli* strains GC138 and HB101 were used as positive and negative controls, respectively (dos Santos et al., 2017). After ON incubation at 36°C, the strains producing bacteriocin appeared surrounded by an inhibition zone of the indicator strain in TYE and NA. As a counterproof, the presence of trypsin in the TYE-T would abolish the bacteriocin effect, resulting in non-inhibited growth of the indicator strain by the bacteriocin-producer strains.

The strains that were positive in the assay were submitted to PCR to evaluate whether the bacteriocin that they produced belongs to bacteriocin types B, E1, E2, Ia, Ib, M, and microcin V using primers (Supplementary Table S1) and conditions described by Šmajs et al. (2010).

### 2.3 Hemolytic activity

The hemolytic activity was assayed by inoculating 3 μL of an ON TSB culture in Columbia agar medium (Kasvi, Pinhais, PR) supplemented with 5% (v:v) of washed and defibrinated sheep blood and 10 mM CaCl_2_ (Beutin et al., 1989). Hemolysis was detected after 3 h and 24 h of incubation at 37°C by a clear zone surrounding the inoculated strain. *E. coli* strains U4-41 and EDL 933 were the positive controls for alpha-hemolysin and enterohemolysin production, respectively. *Klebsiella* spp. was the negative control. The presence of the *hlyA* and *ehxA* genes was searched by PCR (Supplementary Table S2) for all hemolytic strains as previously described (Schmidt et al., 1995; Yamamoto et al., 1995; Johnson and Stell, 2000).

### 2.4 Serum resistance assay

The serum resistance assay was done using a commercially acquired pool of human sera (Sigma-Aldrich, MO, United States). A logarithmic phase culture grown in TSB was washed twice using phosphate-buffered saline (PBS). The bacterial concentration was adjusted to 1 × 10^7^ CFU/mL in PBS. The 100 μL of bacterial suspension was mixed with normal serum (1:1 v) and incubated at 37°C for 24 h. Another bacteria-serum mixture was prepared using Complement-inactivated serum obtained by heating at 56°C for 30 min. At 0 and 24 h, 5 μL of the mixtures were plated onto MacConkey agar to detect strains that survived serum bactericidal activity during the evaluated period. The *E. coli* K-12 strain MG1655 was used as a serum-sensitive control, and the strain *E. coli* J96 (ExPEC prototype) was used as a serum-resistant control.

The genes *iss* and *traT,* considered involved in serum resistance, were searched by PCR in all aEPEC strains as previously described (Johnson and Stell, 2000; Dezfulian et al., 2003) using specific primers (Supplementary Table S2).

### 2.5 Phylogenetic origin, ExPEC and UPEC molecular classification, presence of pathogenicity islands, and clonal relationship

The phylogenetic origin of aEPEC strains was determined using the Clermont quadruplex PCR scheme (Clermont et al., 2013) followed by confirmation (for phylogroups A, C, D, and E), which enables the classification of *E. coli* into one of seven phylogroups (A, B1, B2, C, D, E, and F).

The aEPEC strains were molecularly classified as ExPEC by the presence of at least two genes among *papC*, *afaBC*III, *sfaDE*, *kpsMT*II, and *iutA* (Johnson et al., 2003), and as UPEC when presenting at least three among *vat, chuA, fyuA,* and *yfcV* (Spurbeck et al., 2012). The PCR reactions were performed using primers and conditions described in Supplementary Table S3 (Le Bouguenec et al., 1992; Johnson et al., 1997; Johnson and Stell, 2000; Spurbeck et al., 2012).

Eight pathogenicity islands (PAIs) present in ExPEC prototype strains CFT073 (I_CFT073_ and II_CFT073_), J96 (I_J96_ and II_J96_), and 536 (I_536_, II_536_, III_536_, and IV_536_) were searched using primers and conditions (Supplementary Table S4) described by Sabaté et al. (2006) with minor modifications (da Silva et al., 2017).

The clonal relationship of aEPEC strains was assessed by RAPD using primers 1247 (AAGAGCCCGT) and 1283 (GCGATCCCCA) as previously described for *E. coli* (Nielsen et al., 2014). To determine a clonal relationship, the amplification pattern obtained for both primers was evaluated by the BioNumerics program version 7.6.3 (Applied Maths, Sint-Martens-Latem, Belgium). The similarity of 90% among the amplification profiles for both primers was established as a cutoff to determine the clonal relationship among the strains evaluated.

All PCR reactions were performed using GoTaq® Green Master Mix (Promega, WI, United States) and freshly boiled bacteria as templates. The PCR products were evaluated after electrophoresis in agarose gel.

### 2.6 Characterization of strains presenting the aEPEC/ExPEC and aEPEC/UPEC hybrid genotypes

#### 2.6.1 Whole genome sequencing of the potentially hybrid aEPEC strains

The aEPEC strains 0811–4, 1551–3, 2071–1, and 3712–3, presenting molecular characteristics of the ExPEC pathotype (including UPEC), here designated as hybrid aEPEC, were chosen to have their genome sequenced by the Microbes NG sequencing service^1^ as previously published (Santos et al., 2021). Briefly, the genomes were sequenced using the Illumina sequencing platform to obtain 2 × 250 bp paired reads. Each genome was assembled using the SPAdes software (version 3.7), and contigs were annotated using Prokka (version 1.11). Genome analyses were conducted at the Center for Genomic Epidemiology (CGE)^2^ using their services for the identification of virulence genes (VirulenceFinder version 2.0) (Joensen et al., 2014; Malberg Tetzschner et al., 2020), serotype (SeroTypeFinder version 2.0) (Camacho et al., 2009; Joensen et al., 2015), antibiotic resistance genes (ResFinder version 4.1) (Zankari et al., 2017; Bortolaia et al., 2020), plasmids (PlasmidFinder version 2.0) (Carattoli et al., 2014), *fumC* and *fimH* typing (CHTyper version 1.0) (Roer et al., 2018), and sequence type determination (MLST version 2.0) (Wirth et al., 2006), following the Warwick scheme.

The presence of SNPs and number of SNP clusters in clonal-related strains were evaluated using the pathogen detection tool at the National Database of Antibiotic Resistant Organisms (NDARO)^3^ (Cherry, 2017).

#### 2.6.2 Clonal relationship between hybrid aEPEC strains and EPEC/EHEC clonal groups

Two phylogenetic trees were built to evaluate the clonal relationship of the hybrid aEPEC/ExPEC strains. One tree, using *E. coli* genomes previously published (Hazen et al., 2016; Ingle et al., 2016; Hernandes et al., 2020), was used to identify to which EPEC/EHEC clonal group the hybrid aEPEC strains belong. The other tree was built using the genomes of the aEPEC strains 0811–4, 1551–3, and 3712–3 (this study), and 93 *E. coli* published genomes as previously described (Santos et al., 2021). The 93 *E. coli* public genomes were selected using the Similar Genome Finder Service and the Phylogenetic Tree Building Service at Bacterial and Viral Bioinformatics Resource Center–BV-BRC^4^ (formerly PATRIC) (Wattam et al., 2017) and details of the strains used are available in Supplementary Table S5. The genomes were downloaded from NCBI and analyzed using VirulenceFinder to determine the virulence genes considered hallmarks of EPEC, STEC, ExPEC, and UPEC. The trees’ final layout was finalized in iTOL v6 (Letunic and Bork, 2021).

### 2.7 Virulence in the *Galleria mellonella* model

The virulence of aEPEC strains carrying ExPEC characteristics was investigated “*in vivo*” using the *Galleria mellonella* model following procedures previously described (Santos et al., 2020b). An inoculum of 10 μL of the bacterial suspension containing 1 × 10^7^ CFU/mL was injected into the larvae prolegs using a hypodermic insulin syringe with a 31-gauge needle (Uniqmed, Chungcheongnam-do, KOR). The CFU injected inoculum was confirmed by plate count. The tests were performed in three independent assays, using five larvae for each strain tested. The *E. coli* strains J96 and MG1655 were used as positive and negative controls, respectively, and PBS injections were used as the procedure control. The *E. coli* JPN15, an EAF plasmid-cured EPEC strain derivative from O127: H6 strain E2348/69 (Levine et al., 1985) was used as an aEPEC control.

### 2.8 Statistical analyses

The Kaplan–Meier survival curve was used for survival analysis, and the differences between the groups were determined by the log-rank (Mantel-Cox) and Gehan-Breslow-Wilcoxon tests. The threshold for statistical significance was a *p*-value < 0.05. All analyses were performed using GraphPad Prism 9.2 (GraphPad Software, LLC).

## 3 Results

### 3.1 Frequency of bacteriocinogenic and hemolytic strains among aEPEC

In this work, we have searched for the production of bacteriocins, hemolysins, and serum resistance, which are recognized as contributors to the ExPEC virulence pathotype (Johnson and Russo, 2018) in aEPEC strains.

Production of bacteriocins in TYE and NA was detected in 24 (21.6%) aEPEC strains, with results of both media in accordance. All were confirmed producers by the test using the same media supplemented with trypsin. The bacteriocinogenic strains were submitted to molecular typing, and the bacteriocin of 16 (67%) were successfully genetically typed. The bacteriocins Ia and Ib were the most frequently identified and occurred together in six strains, while microcin V was identified in only one strain (Table 1). The bacteriocin types E1 and E2 were identified in three strains each, and the bacteriocin M was identified in another three strains, co-occurring with bacteriocin B in two of them (Table 1).

**Table 1:** Type of bacteriocin identified in 24 aEPEC bacteriocinogenic strains.

Type of bacteriocin	Occurrence (%)
Ia and Ib	6 (25)
E1	3 (12.5)
E2	3 (12.5)
M and B	2 (8.3)
M	1 (4.2)
V	1 (4.2)
Not typed	8 (33.3)

Twenty-one (18.9%) aEPEC strains were hemolytic when cultivated in Blood agar. For three aEPEC strains, hemolysis was detected after 3 h of incubation and in the others after 6 or 18 h of incubation. The hemolytic strains were evaluated by PCR for the presence of the genes *hlyA* (*α*-hemolysin) and *ehxA* (enterohemolysin), confirming the occurrence of one of these genes in 19 strains. Seventeen of the hemolytic aEPEC strains harbored *hlyA* and two *ehxA*. The remaining two strains, although hemolytic, were not detectable by the molecular method used.

### 3.2 aEPEC strains can survive in human sera

The ability to survive the action of the Complement system active in normal human serum is a main virulence property that enables extraintestinal pathogens to succeed in passing through the bloodstream and gain access to extraintestinal sites.

The majority of aEPEC strains (n = 95, 85.6%) studied were viable after the 24-h challenge in the presence of human sera at a concentration of 50% in PBS. Among the resistant strains, only 13 survived during the challenge, reaching a low concentration in human sera, while the others kept high concentrations at the end of the assay. Sixteen strains were susceptible to human sera effects and did not survive the challenge. Thirty-eight (34.2%) strains were positive for *traT,* and only four of them did not display the serum-resistant phenotype. No strain was positive for the genes *iss* and *kpsMT*II with the primers used.

### 3.3 The phylogenetic origin, the putative presence of ExPEC PAIs, and genetic virulence markers in aEPEC

The phylogenetic origin of the 111 aEPEC strains as determined by PCR revealed that phylogroup B1 was the most frequent (54 strains—48.7%), followed by groups A (32 strains—28.8%), B2 (15 strains—13.5%), and E (9 strains—8.1%). Phylogroup D presented only one strain (0.9%). No strain from phylogroups C and F was identified. No difference was detected in phylogenetic distribution comparing strains isolated from cases and controls (Supplementary Table S6).

Searching the PAIs by PCR, it was found that about 55% of the aEPEC strains presented PAIs originally described in UPEC. Table 2 shows the distribution of these PAIs among the aEPEC strains according to their phylogroups. Five out of eight PAI markers searched were found in aEPEC, with only one PAI occurring in each strain, except for PAIs IV_536,_ and I_CFT073_, which appeared combined in two strains, and PAIs II_536,_ and I_J96_ in another two (Table 2). The PAIs most frequently detected were IV_536_ (38 strains—34.2%), I_J96_ (10 strains—9%), and I_CFT073_ (9 strains—8.1%), comprising 55 out of 61 PAI-positive strains. No strain presented PAI markers for islands II_J96_, I_536_, and III_536_.

**Table 2:** Distribution of PAI markers in 111 aEPEC strains according to their phylogenetic origin.

Phylogroup (n)	Strains presenting PAI-markers [n (%)]	PAIs’ profile^a^	N (%) of strains^b^
A (32)	21 (65.6)	IV_536,_ I_CFT073_	1 (4.8)
		IV_536_	14 (66.7)
		II_536_	3 (14.3)
		I_CFT073_	1 (4.8)
		II_CFT073_	2 (9.5)
B1 (54)	33 (61.1)	II_536,_ I_J96_	2 (6.1)
		IV_536_	21 (63.6)
		I_CFT073_	2 (6.1)
		I_J96_	8 (24.2)
B2 (15)	6 (40.0)	IV_536,_ I_CFT073_	1 (16.7)
		I_CFT073_	4 (66.7)
		IV_536_	1 (16.7)
D (1)	0 (0)	–	–
E (9)	1 (11.1)	II_CFT073_	1 (100)
All groups (111)	61 (54.9)		

a PAI designation: I_536_, IV_536_, Pathogenicity Islands I and IV from UPEC strain 536; I_CFT073_, II_CFT073_, Pathogenicity Islands I and II from UPEC strain CFT073; I_J96_, Pathogenicity Island I from UPEC strain J96.

b The percentage refers to the total of strains presenting PAIs in the phylogroup.

Virulence factors related to ExPEC intrinsic virulence (*afa/dra, sfa/foc, pap, kpsMT*II*, iuc/iut*) and uropathogenic potential (*vat, fyuA, chuA, yfcV*) were searched. Many aEPEC strains presented at least one of these virulence genetic markers that are considered the hallmark of the capacity of the strains to cause systemic or urinary tract infections in murine models. Although many of these genes were spread in a variety of aEPEC strains (Table 3), only five could be classified as ExPEC (strains 0811–4, 2071–1, 1482–11, 2332–7, and 1551–3), and another one as UPEC (strain 3712–3) (Table 3), highlighting the existence of hybrid genotypes among aEPEC strains.

**Table 3:** Frequency of virulence genetic markers^a^, and molecular classification of ExPEC and UPEC in 111 aEPEC strains.

Virulence trait	Genetic marker	n (%)
Iron acquisition system	*fyuA*	38 (34.2)
	*chuA*	25 (22.5)
	*iutA*	25 (22.5)
Adhesin	*yfcV*	12 (10.8)
	*afaBC*	5 (4.5)
	*papC*	0
	*sfaDE*	0
Toxin	*vat*	2 (1.8)
Protectin	*kpsMTII*	0

Molecular classification	Criteria used^b^	
ExPEC	Presence of at least two of: *afa, iutA, kpsMTII, pap,* and *sfa*	5 (4.5)
UPEC	Presence of at least three of: *chuA*, *fyuA*, *vat*, and *yfcV*	1 (0.9)

a Only those proposed to define ExPEC and UPEC^b^.

b The genes searched for the molecular criteria represent: *afa* - afimbrial adhesin, *pap* - P fimbriae, *sfa* - S fimbriae, *kpsMTII* - capsule belonging to capsular group 2, *iuc/iutA* - aerobactin, *yfcV* - YFC fimbriae, *vat* - vacuolating autotransporter toxin, *fyuA* - yersiniabactin, and *chuA* - heme-binding protein (Johnson et al., 2003; Spurbeck et al., 2012).

Underline means the virulence factors related to each gene.

Regarding the presence of VFs associated with PAIs, except for *fyuA* and PAI IV, the other VFs did not hold this association in most strains. This was pointed out by the absence or rare co-occurrence of *pap* (related to PAI II_CFT073_, PAI II_536_, and PAI I_J96_)*, sfa* (related to PAI III_536_), *kps* (related to PAI I_CFT073_), and *hlyA* (related to PAI I_J96_) among the aEPEC strains evaluated.

Table 4 presents a summary of the virulence properties that were detected in the aEPEC strains studied, comparing the frequency of each property by phylogroup.

**Table 4:** Number (%) of aEPEC strains presenting ExPEC virulence properties according to phylogroup.

Virulence properties	Phylogroup (n)					
	A(32)	B1(54)	B2(15)	D(1)	E(9)	TOTAL(111)
	n (%)	n (%)	n (%)	n (%)	n (%)	n (%)
Bacteriocin production	3 (9.4)	12 (22.2)	0	0	9 (100)	24 (21.6)
Hemolysin production	10 (31.2)	11 (20.4)	0	0	0	21 (18.9)
Serum resistance	25 (78.1)	47 (87.0)	13 (86.7)	1 (100)	9 (100)	95 (85.6)
PAI markers	21 (65.6)	33 (61.1)	6 (40.0)	0	1 (11.1)	61 (54.9)
ExPEC defining genes^a^	0	5 (9.2)	0	0	0	5 (4.5)
UPEC defining genes^b^	0	0	1 (6.7)	0	0	1 (0.9)

a Presence of at least two among *pap*, *sfa*, *afa*, *iuc/iutA*, and *kpsMTII*.

b Presence of at least three among *yfcV*, *fyuA*, *chuA* and *vat*.

Not surprisingly, the occurrence of the virulence properties followed the phylogenetic distribution, and most of the traits occurred in phylogroups A and B1, which are the most frequent among aEPEC strains. Serum-resistant strains were identified in all phylogroups, with phylogroup E concentrating the higher frequency of resistant strains than phylogroups A, B1, and B2 (Table 4). The five aEPEC/ExPEC hybrid strains (0811–4, 2071–1, 1482–11, 2332–7, and 1551–3) belonged to phylogroup B1, and the hybrid aEPEC/UPEC (3712–3) belonged to phylogroup B2.

### 3.4 Clonal relationship and genomic sequencing of hybrid aEPEC strains

The RAPD PCR methodology was used to evaluate the clonal relationship among the aEPEC strains, especially the six aEPEC strains identified as possessing hybrid genotypes, according to the amplification obtained using the primers 1247 and 1283 and considering a cutoff of 90% of similarity for both PCRs. RAPD highlighted the high heterogenicity of the aEPEC strains evaluated, with most not being grouped in a cluster (Supplementary Figure S1).

**Figure 1:**
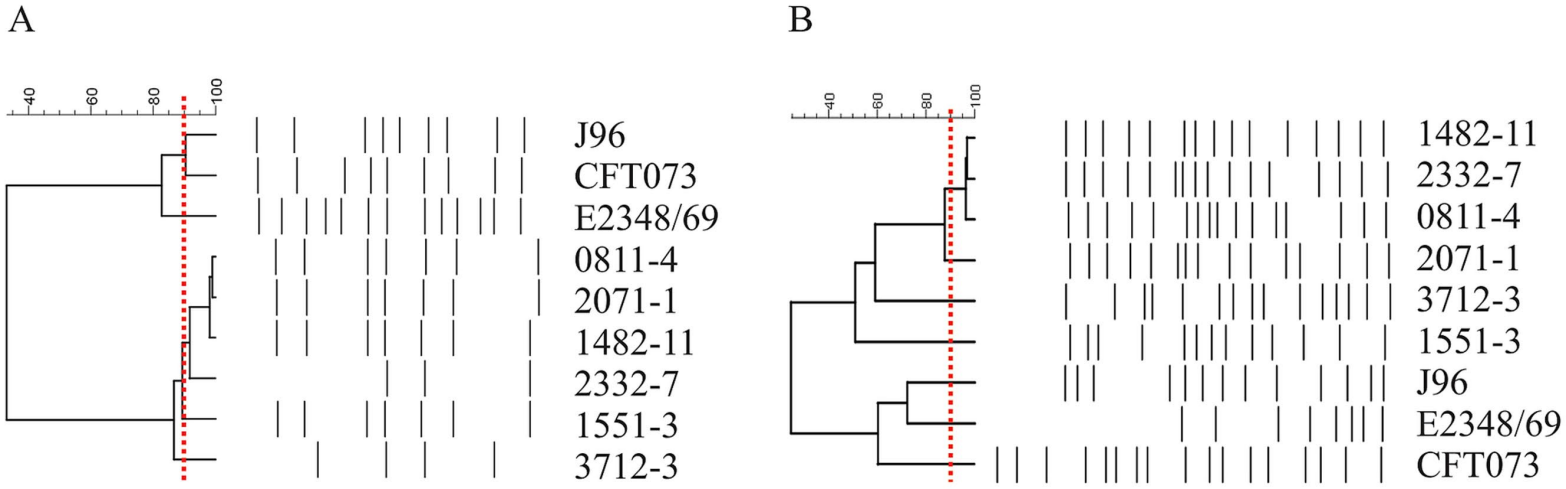
Dendrogram of six aEPEC presenting hybrid genotypes. Amplification profiles obtained in RAPD-PCR using primer 1247 **(A)** and primer 1283 **(B)** were evaluated by the BioNumerics and used to build the dendrogram. Note the proximity among strains 0811–4, 2071–1, 1482–11, and 2332–7. Strains 1551–3 and 3712–3 were less correlated. The red dashed line represents the cutoff of 90% similarity in the amplification pattern used to determine the clonality of the strains.

The hybrid aEPEC strains 0811–4, 1482–11, and 2332–7 are closely related, indicating a clonal relationship. The strain 2071–1 presents a similarity of >95% using primer 1247 but a similarity between 87 and 90% with primer 1283, which raised doubts about its relationship with the other three aEPEC strains. On the other hand, 1551–3 and 3712–3 were not closely related either to each other, or to the first group (Figure 1).

Considering the clonal relationship among 0811–4, 1482–11, and 2332–7, the aEPEC 0811–4 and the more distant strain 2071–1 were chosen to represent this group, being submitted together with 1551–3 and 3712–3 to genome sequencing.

The genome sequences revealed that the strains had predicted genome sizes ranging from 4,784,160 to 5,328,743 bp, with strains 0811–4 and 2071–1 displaying larger predicted genome sizes (Table 5). Three STs were identified among the sequenced strains: 0811–4 and 2071–1, which were clonally related, as suggested by RAPD, belong to ST381; strain 1551–5 belongs to ST29, and strain 3712–3 to ST1092 (Table 5).

**Table 5:** Genome predicted size, metrics of the WGS, and MLST of aEPEC hybrid strains evaluated in this study.

Strain N°	Genome size (bp)	Contigs	L50	N50	MLST-phylogroup
0811–4	5,328,743	338	15	134,776	ST381-B1
2071–1	5,316,170	437	18	102,511	ST381-B1
1551–5	5,066,720	281	15	114,652	ST29-B1
3712–3	4,784,160	57	7	257,658	ST1092-B2

A phylogenetic analysis conducted with 0811–4 and 2071–1 confirmed that they are clonally related (Supplementary Figure S2). Additionally, the pathogen detection tool showed that they belong to the same SNP cluster PDS000052783.4, differing by only 18 SNPs. Therefore, only 0811–4 was taken as a reference for the four aEPEC/ExPEC strains from ST381 for other evaluations.

**Figure 2:**
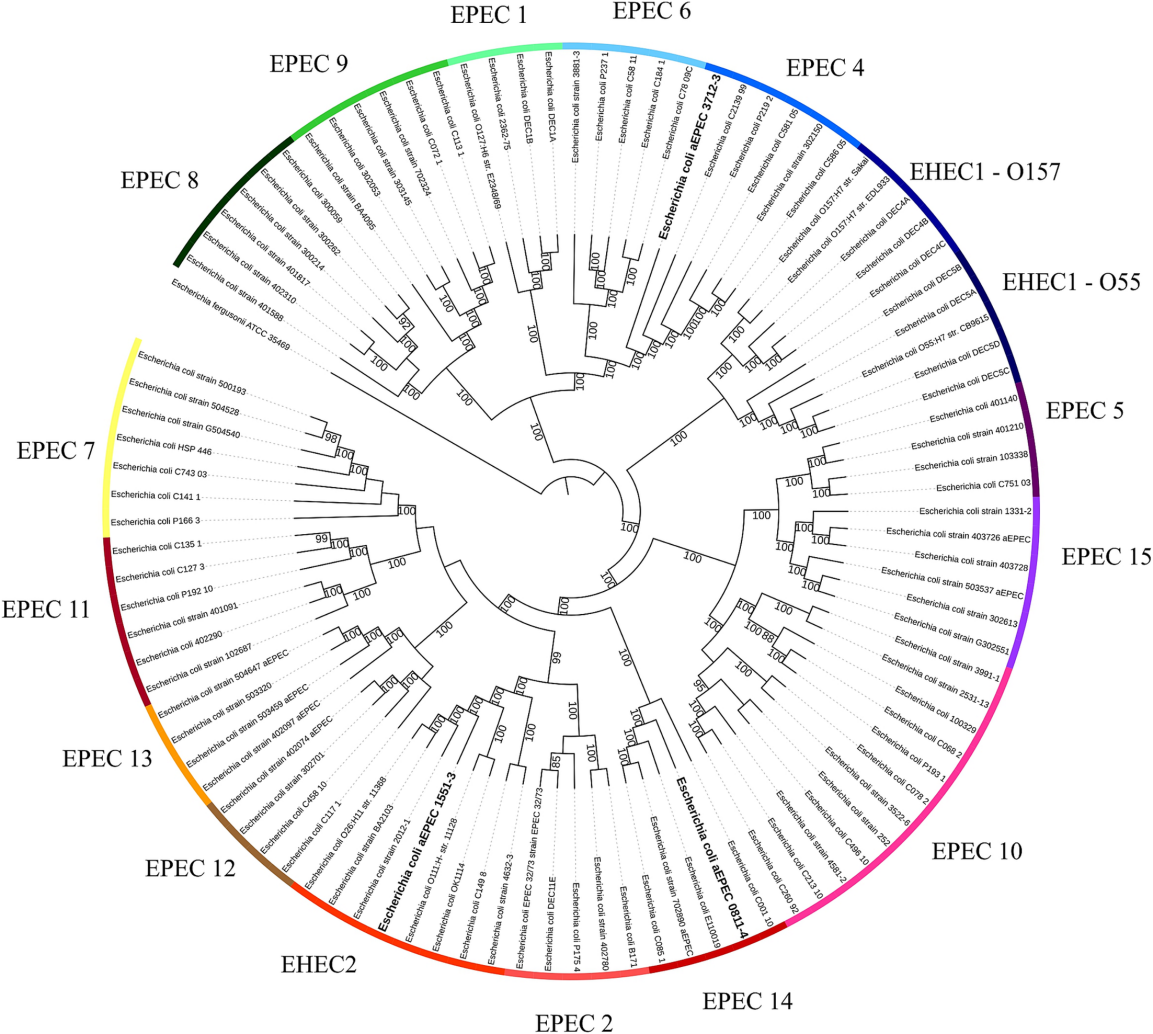
EPEC and EHEC clonal lineages. The whole genome sequence of the hybrid strains was evaluated to identify to which EPEC/EHEC lineage they belong. Each one of the hybrids belongs to a different clonal lineage: EPEC 4 (3712–3), EPEC 14 (0811–4), and EHEC 2 (1551–3). The phylogenetic tree was built with the codon tree method, evaluating 1,000 single-copy CDS (amino acid and nucleic acid sequences) in an RA × ML matrix, using the genomes of previously published strains (Hazen et al., 2016; Hernandes et al., 2020) and the hybrids aEPEC/ExPEC (0811–4 and 1551–3) and aEPEC/UPEC (3712–3). Each strip segment represents one of the EPEC or EHEC clonal lineages. The hybrid strains are highlighted in bold. *Escherichia fergusonii* was used as an out-group to root the tree. Bootstrap above 80 was displayed in the tree.

A second phylogenetic analysis, including all hybrid strains, showed that these three strains belong to different EPEC/EHEC clonal groups, with 1551–3 belonging to the EHEC 2 clonal group, 0811–4 to EPEC 14, and 3712–3 to EPEC 4 (Figure 2).

**Figure 3:**
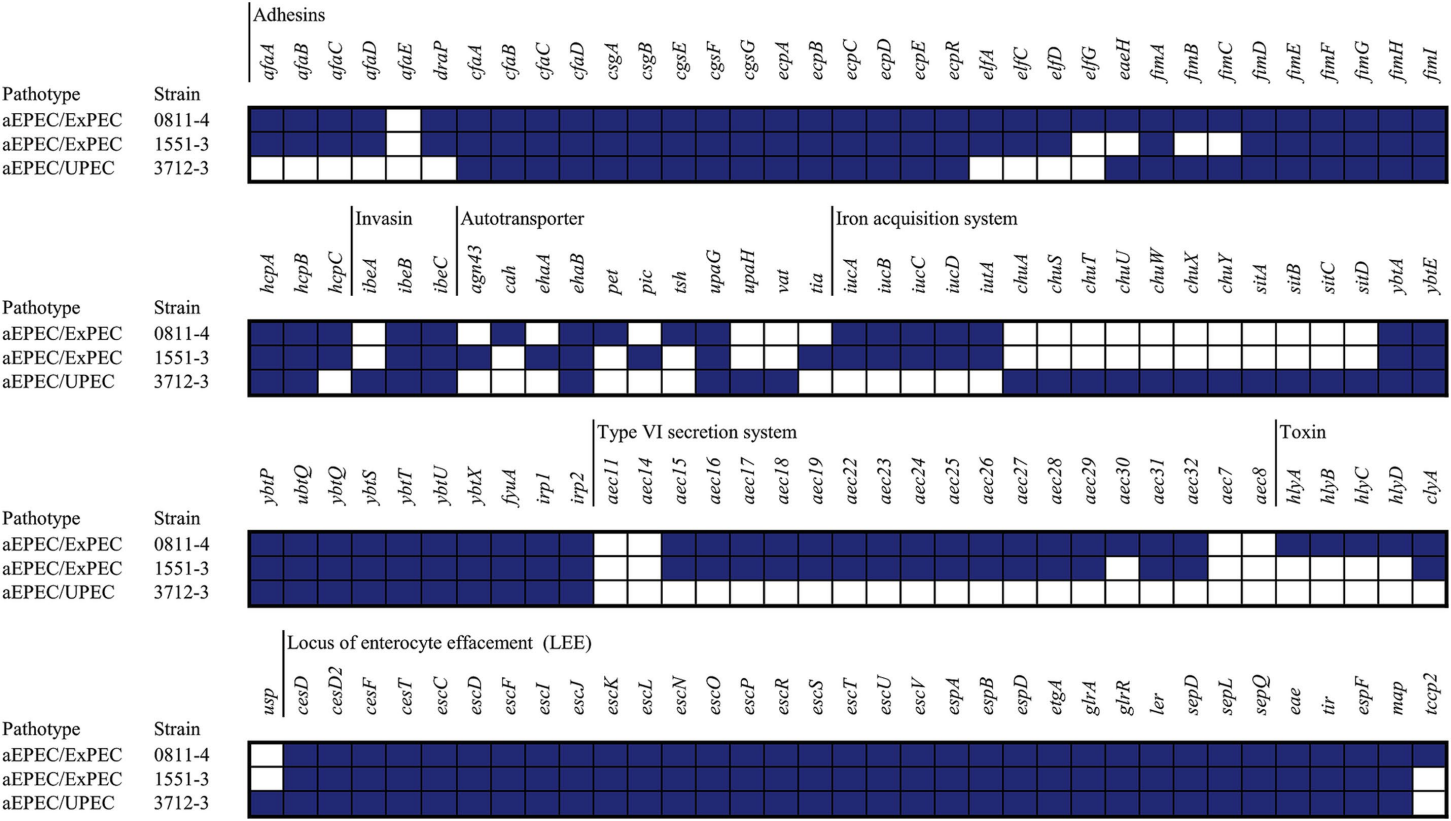
VFs of hybrid aEPEC identified. The VFs identified using VFDB were used to build this figure. The filled squares represent the presence of the genes, while the empty squares represent the absence. Only genes identified in at least one strain were displayed in this figure.

The assembled genomes were submitted to VFDB, and the VF content was compared (Figure 3). It is noticeable that the VF content of the strains is slightly different, although 0811–4 and 1551–3 shared many VFs. In common, besides the LEE, all strains bear the genes related to the expression of CFA/I, ECP, curli, EhaB, UpaG, and Yersiniabactin (Figure 3). In addition, a diverse combination of auto transporters, including SPATES (Vat, Tsh, and Pic) and iron acquisition systems (Aerobactin, *Salmonella* iron transport system, and Heme receptor) was found (Figure 3). Additionally, the profile of VF genes used for molecular classification was identified as complete, including all the genes of operons, and, except for 0811–4, the strains did not share the intimin type or serotype. Strain 0811–3 harbors intimin type epsilon-1, while 1551–3 harbors epsilon-2, and 3712–3 intimin type zeta (Table 6).

**Table 6:** Serotype, intimin type, and molecular classification of hybrid aEPEC strains.

Strain	Serotype		Intimin variant	Molecular classification
	Phenotype	Genotype		
0811–4	NT: H-	O126/O186: H9	ε1 (epsilon 01)	aEPEC/ExPEC
2071–1	NT: H-	O126/O186: H9	ε1 (epsilon 01)	aEPEC/ExPEC
1551–3	O26: H-	O26: H8	ε2 (epsilon 02)	aEPEC/ExPEC
3712–3	NT: H29/H31	O179: H31	ζ (zeta)	aEPEC/UPEC

The finding of hybrid aEPEC strains presenting UPEC or ExPEC molecular signatures drove us to search for similar genomic characteristics in a larger group of aEPEC strains that have their genomes accessible in the NCBI. The similar genomes, identified using MASH/MinHash, helped the construction of an additional phylogenetic tree that was added with the VFs used to molecular define the EPEC, EHEC, ExPEC, and UPEC pathotypes.

**Figure 4:**
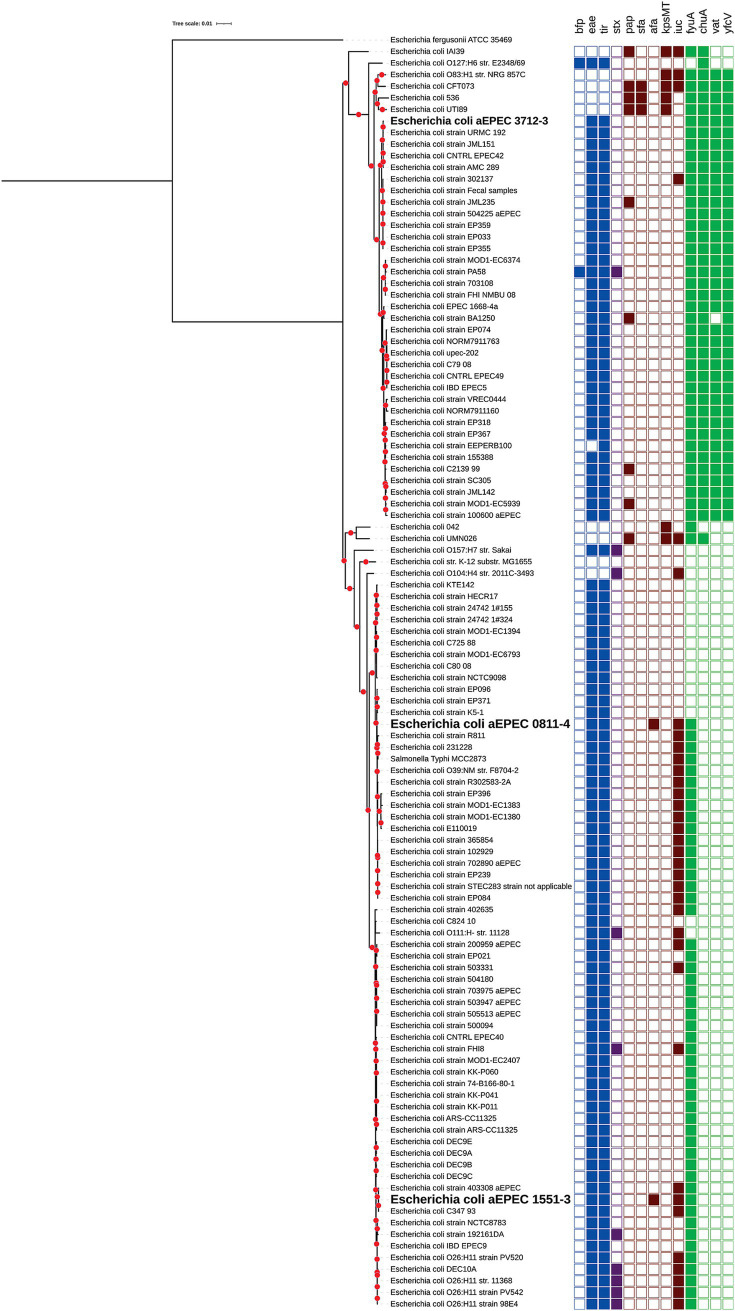
Phylogenetic tree comparing the VFs of the aEPEC hybrid strains and 93 *E. coli* strains with similar genomes available at NCBI. All *E. coli* strains displaying a similar genome with aEPEC/UPEC strain 3712–3 also harbor VFs related to the UPEC molecular classification. The VFs used for strain comparison were those associated with the molecular classification of EPEC (in blue: *bfpA, eae,* and *tir*), STEC (in purple: *stx*), ExPEC (in red: *pap, sfa, afa, kpsMT,* and *iuc*), and UPEC (in green: *fyuA, chuA, vat,* and *yfcV*) and identified by VirulenceFinder. Bootstrap ≥ 80 were displayed in the tree as red dots. The three hybrid strains sequenced in the present study were highlighted in bold.

The tree’s analyses showed that all EPEC genomes closely related to 3712–3 were also hybrid aEPEC or EHEC bearing at least three of the required VFs to be classified as UPEC (Figure 4), suggesting that at least some of the strains that compose the EPEC4 clonal group (see Figure 2) are potentially extraintestinal pathogens. In contrast, the other two hybrid aEPEC in this study were unique in presenting ExPEC VFs within their clusters. Yet, it is worth mentioning that 0811–4 is clonal related with other three hybrid aEPEC strains (1482–11, 2071–1, and 2332–7) evaluated in the present study, pointing out that the spread of aEPEC/ExPEC hybrid genotypes is a reality among diarrheagenic strains isolated in Brazil.

### 3.5 The hybrid aEPEC tend to be virulent in the *G. mellonella* model

In this study, the *G. mellonella* model was used to evaluate the pathogenic potential of three of those above-mentioned aEPEC strains that presented genotypic and phenotypic characteristics of ExPEC, meaning that they represented a hybrid class of pathogenic strains.

**Figure 5:**
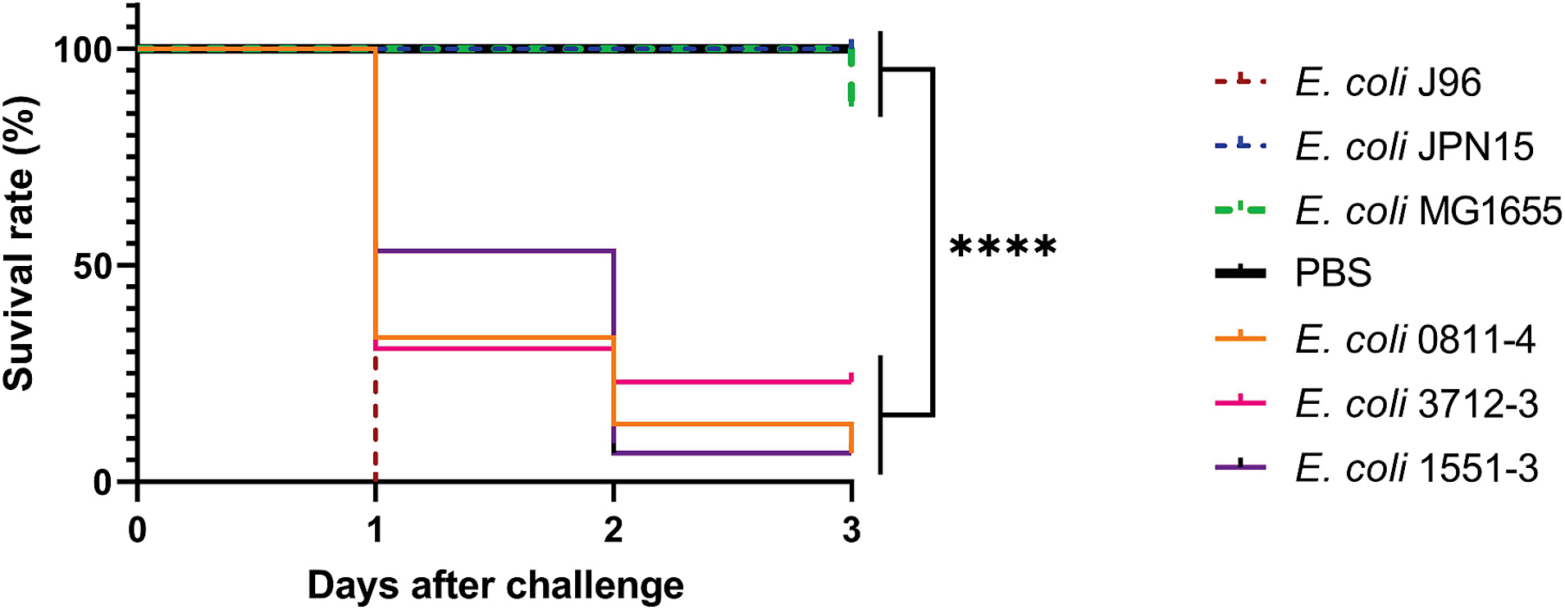
Virulence in *G. mellonella* was assessed for one hybrid strain belonging to each RAPD type using the Kaplan–Meier survival curve. Each larva was injected with 1 × 10^5^ CFU, and incubated at 37°C for 3 days. The ExPEC prototype strain J96 was used as a positive control, and PBS as the procedure control. ****p < 0.0001. Strains JPN15 and MG1655 were used as negative controls.

The results presented in Figure 5 show that all hybrid strains evaluated (aEPEC/ExPEC 0811–4 and 1551–3, and aEPEC/UPEC 3712–3) were capable of killing more larvae than the negative-control strains MG1655 and JPN15, a variant of prototype EPEC strain E2348/69 devoid of the virulence plasmid. However, the survival rate of larvae for the three hybrid strains was higher when compared to the prototype J96, being higher for 1551–3 (*p =* 0.0012) than for 0811–4 and 3712–3 (*p =* 0.01 and 0.02, respectively). There was no statistical difference in virulence among the three hybrid strains in this model (*p* < 0.05). These data suggest that the inoculum of 1 × 10^5^ CFU per larvae can differentiate diarrheagenic EPEC strains from those harboring a hybrid genotype. However, the inoculum used cannot distinguish the genomic diversity of the aEPEC hybrid strains.

## 4 Discussion

Atypical EPEC is the main *E. coli* pathotype responsible for diarrhea in Brazil (Afset et al., 2004; Aranda et al., 2004; Liebchen et al., 2011; Ori et al., 2019; Beraldo et al., 2023) and other low- and middle-income countries (Jarquin et al., 2022; Kara et al., 2022; Yamani and Elhadi, 2022). Since this pathotype was defined, it has been essentially recognized as an intestinal pathogen. This concept started to be challenged by an increasing number of reports referring to the isolation of IPEC of various pathotypes from the bloodstream.

*E. coli* is known to be a bacterium permissive for the acquisition and stable maintenance of foreign DNA brought by various mechanisms that mediate horizontal gene transfer (HGT) (Desvaux et al., 2020). Such genome flexibility helped to understand the existence of strains presenting mixed virulence genes that used to be characteristic of specific pathotypes.

In 2015, Kessler et al. (2015) reported one case of an inpatient with diarrhea that evolved to bacteremia with multiorgan dysfunction due to an ExPEC bearing EPEC VFs. Even though it was not the first report regarding the EPEC pathotype causing extraintestinal infections (Bratoeva et al., 1994; Abe et al., 2008; Toval et al., 2014), the study contributed to the increase in the number of studies reporting the presence of EPEC strains in extraintestinal infections (Riveros et al., 2017; Valiatti et al., 2020; Munhoz et al., 2021; Nascimento et al., 2021, 2022; Yousefipour et al., 2023).

The knowledge of those versatile bacteria prompted the scientific community to consider the concept of hybrid pathogenic *E. coli* strains (Santos et al., 2020a) therefore intensifying the studies for a better understanding of their virulence potential and their role as a threat to public health. The importance of hybrid pathogenic *E. coli* bearing IPEC and ExPEC VFs relies on the possibility of these diarrheagenic bacteria translocating from the intestinal lumen, crossing the gut barrier, and reaching the bloodstream to cause systemic infection, which poses an increasing risk of patient death (Kessler et al., 2015; Mandomando et al., 2020; Santos et al., 2020a). Considering the WHO reports that inform that most diarrhea-related deaths are due to septic shock (WHO, 2017), it is important to identify what are the pathogens that are associated with both conditions and, therefore, require additional surveillance and better diagnosis to avoid fatal outcomes mainly among children.

In the present work, we studied 111 aEPEC strains isolated from patients and asymptomatic carriers, evaluating aspects related to phylogenetic origin, presence of PAIs and VFs associated with the ExPEC pathotype, to assess the extension of sharing of virulence characteristics between these two pathotypes, as well as the existence of putative hybrid pathogens among aEPEC strains.

As various studies (Hazen et al., 2016; Richter et al., 2018; Hernandes et al., 2020) have already demonstrated, the phylogenetic analyses showed that most of the aEPEC strains belonged to phylogroups B1 (48.6%) and A (28.8%).

As many as 61 (54.9%) strains possessed genetic PAI markers first described in UPEC. The presence of PAI markers in all phylogroups of aEPEC, except for phylogroup D, poorly represented by one strain, suggests that the accessibility of the aEPEC population to HGT is independent of its phylogenetic origin. Diverse PAI markers were identified in the sample studied, with PAI IV_536_ being the most prevalent (34.2%), and detected mainly in phylogroups B1, and A.

The PAI IV_536_, also known as High Pathogenicity Island (HPI), harbors the iron uptake system called yersiniabactin. Recent studies demonstrated that yersiniabactin contributes to the intrinsic virulence of extraintestinal pathogens and is involved in the capacity of *E. coli* to kill mice in a murine model of sepsis (Galardini et al., 2020). Besides, it has been used as a molecular marker to identify *E. coli* strains that are potentially uropathogenic (Spurbeck et al., 2012).

It has to be pointed out that, in our results, the detection of the PAI markers was not in complete agreement with the detection of the virulence genes they were supposed to carry according to the literature, i.e., *hlyA, pap, kpsMTII*, and *iuc* for I_CFT073_, *pap* for II_CFT073_, *hlyA* and *pap* for I_J96_, and *pap* for II_536_ (Dobrindt et al., 2002; Sabaté et al., 2006; Desvaux et al., 2020). The only exception occurred with PAI IV_536_ which was associated with the genes *fyuA* and *irp2* (Dobrindt et al., 2002; Desvaux et al., 2020) in all positive strains. These findings may be due to the presence of incomplete PAIs or even differences in the content of genes they carry. Studies will be addressed to better understand the relationship between the UPEC PAIs and the aEPEC pathotype.

Although more than 50 VFs have been described so far as playing a role in ExPEC virulence (Johnson and Russo, 2018), different studies have shown that it is possible to use a minimum set of VFs to recognize *E. coli* strains as potential agents of extraintestinal infections, regardless of the bacteria isolation source (Picard et al., 1999; Johnson et al., 2000, 2006, 2022; Spurbeck et al., 2012; Wijetunge et al., 2015; Mellata et al., 2018; Galardini et al., 2020). Further refined studies evaluating the bacterial virulence *in vivo* in the murine models for sepsis and urinary tract infection (Johnson et al., 2003; Spurbeck et al., 2012), have demonstrated that two sets of genes would be reliable to molecularly classify *E. coli* that can cause extraintestinal infections in humans (Johnson et al., 2003; Spurbeck et al., 2012). Accordingly, in this study, aEPEC was positive for at least one of all the gene markers used for this molecular classification, except for *kpsMTII*, *papC,* and *sfaDE*. This demonstrates once again that aEPEC frequently shares genetic features responsible for the pathogenicity of ExPEC. Moreover, six aEPEC reunited the right gene combinations to have genotypes identified as hybrid pathogens: five aEPEC/ExPEC, and one aEPEC/UPEC.

Besides the presence of a variety of ExPEC virulence genes, many aEPEC strains were shown to produce bacteriocins and hemolysins which are virulence traits also described as involved in ExPEC virulence, although not exclusively.

The distribution of the virulence properties evaluated among aEPEC in phylogroups showed that their occurrence followed the phylogenetic distribution, occurring more in phylogroups A and B1, which were the most frequent among aEPEC strains. Additionally, the presence of PAI markers and the identification of hybrid strains in phylogroup B1 reinforce the genomic plasticity of strains from these phylogroups, enabling them to receive additional VFs related to extraintestinal pathogenicity, and therefore, prompting them to cause extraintestinal infections.

The resistance to the action of complement present in the human serum is an essential property for pathogens to survive in the bloodstream (Miajlovic and Smith, 2014). Surprisingly, most aEPEC strains (85.6%) survived the serum resistance assay. This is a frequency similar to that found among strains isolated from bloodstream infections in clinical settings (Miajlovic and Smith, 2014), and this frequency is much higher than the one previously reported for fecal strains (Miajlovic and Smith, 2014).

Among the three genes (*kpsMT*II, *traT*, and *iss*) usually related to serum-resistant phenotype and searched in this survey, only *traT* was found in less than 36% of the serum-resistant strains, while *kpsMT*II and *iss* were not detected at all. In regard to the *iss* gene, it has to be mentioned that the primers used to search it are specific to type-1 *iss* sequenced (Dezfulian et al., 2003; Johnson et al., 2008), and therefore, it is not possible to rule out the role of the other variant genes in the serum-resistant phenotype observed among aEPEC strains analyzed. Additionally, some of the aEPEC strains studied harbor genes related to serine-protease autotransporters of Enterobacteriaceae (SPATE) (data not shown). Some of these autotransporter proteins were shown to mediate complement cleavage, resulting in serum resistance (Freire et al., 2022; Correa et al., 2024). Therefore, further studies are required to clarify the resistant phenotype identified in the aEPEC strains studied here.

Overall, considering all the differences between IPEC and ExPEC, including those related to the VFs they harbor (Riley, 2020), the simple passage of IPEC strains through the bloodstream would not pose a problem regarding the disease treatment, given that they are not supposed to possess virulence factors to survive or colonize out of the intestinal tract (Johnson and Russo, 2018; Riley, 2020; Denamur et al., 2021). However, the picture changes if these strains are hybrid pathogenic aEPEC/UPEC and aEPEC/ExPEC like those we have detected in this aEPEC survey. In this case, infected patients, mainly children, would be at higher risk of developing extraintestinal infections after colonization of the intestinal tract, which could be either symptomatic or asymptomatic. Riveros et al. (2017), evaluated bacteremia strains isolated from children under 2 years old in Peru and identified diverse IPEC pathotypes, including aEPEC, among the isolates. So far, we are not aware of reports in Brazil about comprehensive epidemiological studies evaluating the presence of IPEC strains carrying ExPEC virulence genes isolated from the bloodstream of children. Nevertheless, there is a report (Liberatore et al., 2011) of an aEPEC isolate, not genotypically hybrid, but displaying the serum-resistant phenotype, which could translocate the intestinal barrier in an experimental translocation model in rats (Liberatore et al., 2011). After the intestinal translocation, the strain was recovered from diverse organs and caused tissue hypoperfusion (Liberatore et al., 2011). This data indicates that among the strains that present serum resistance but lack known virulence markers, there are strains with extraintestinal pathogenic potential that have not yet been adequately evaluated and can potentially cause extraintestinal infections, especially in young children.

The pathogenic potential of three hybrid strains studied in this work (aEPEC/ExPEC 0811–4, 1551–3, and aEPEC/UPEC 3712–3) was proven in the *G. mellonella* model, which has been reported as reliable to assess the virulence of ExPEC (Alghoribi et al., 2014; Williamson et al., 2014; Ciesielczuk et al., 2015; Pereira et al., 2018). The three strains were lethal in that model, although not as efficiently as the positive ExPEC control *E. coli* J96. However, they were significantly more lethal than the non-hybrid aEPEC control, strain JPN15, and the non-pathogenic control, *E. coli* MG 1655.

The genome comparative analysis of the three hybrid strains 0811–4, 1551–3, and 3712–3, showed that they are not phylogenetically related, and accordingly, they belong to different EPEC/EHEC clonal groups, namely EPEC14, EHEC2, and EPEC4, respectively. Corroborating the above analysis, the three hybrid strains belong to different serotypes and sequence types, and all present a complete PAI LEE - the pathogenicity island of EPEC, but display different variants of intimin and its receptor.

Interestingly, the phylogenetic analyses with additional *E. coli* genomes showed that all EPEC strains closely related to 3712–3 are hybrid EPEC/UPEC and bear at least three of the required VFs to be classified as UPEC, suggesting that part of the strains that compose the EPEC4 clonal group are potentially extraintestinal pathogens. In accordance, a few strains identified in this cluster were isolated from extraintestinal infections. Curiously, two other strains isolated in Brazil, PA58, and BA1250, previously published (Gioia-Di Chiacchio et al., 2018; Munhoz et al., 2021), also belong to this cluster. Strain BA1250 was isolated from a diarrhea case in a different geographical region (Munhoz et al., 2021), and strain PA58 was a typical EPEC/Shiga-toxin producer *E. coli* (STEC) hetero-pathogenic strain isolated from a bird (Gioia-Di Chiacchio et al., 2018) that also carries UPEC molecular markers. The other two strains, 0811–4 and 1551–3, are the only strains that bear ExPEC VFs in their clusters (Figure 4). However, it is important to mention that 0811–4 has a clonal relationship with the other three aEPEC strains evaluated in the present study, pointing out that this hybrid genotype is present, and somehow often, among aEPEC strains isolated in São Paulo, Brazil.

Finally, the data herein report the detection of various ExPEC virulence attributes in a comprehensive collection of aEPEC strains. In six out of 111 aEPEC strains studied, it was found the genes fulfilling the molecular criteria for their classification as aEPEC/ExPEC or aEPEC/UPEC hybrid pathogens. In addition, these hybrid pathogens isolated from diarrheal cases were proven to be virulent in an *in vivo G. mellonella* model for ExPEC, suggesting that they can express both intestinal and extraintestinal virulence determinants in humans, turning the consequences of their infection and colonization more severe, and potentially life-threatening.

## Data Availability

The datasets presented in this study can be found in online repositories. The names of the repository/repositories and accession number(s) can be found at: https://www.ncbi.nlm.nih.gov/bioproject/PRJNA871776/.

## Footnotes

^1^ https://microbesng.com

^2^ http://genomicepidemiology.org/

^3^ https://www.ncbi.nlm.nih.gov/pathogens/

^4^ https://www.bv-brc.org/
