# Supplementary material for: The diverse virulence potential of atypical enteropathogenic Escherichia coli isolated from diarrhea: the emergence of a hybrid pathotype?
Source: Front Microbiol. 2025 Jun 6;16:1599350. doi: 10.3389/fmicb.2025.1599350 (PMC12179130; doi:10.3389/fmicb.2025.1599350)
Supplement: Supplementary file 1 [file Data_Sheet_1.PDF]

Supplementary Material

The diverse virulence potential of atypical enteropathogenic *Escherichia coli* isolated from diarrhea. Emergence of an hybrid pathotype?

Ana C. M. Santos [1, 2 ¶], Roberta S. Silva [1 ¶], Mônica A. M. Vieira[2], Cristina V. Niero [3], Matheus S. F. Ribeiro [3], Beatriz E. C. Guth[1], Tânia A. T. Gomes [2], Rosa M. Silva [1*].

Table S1 – Primers and conditions for detection of the colicin and microcin genes

Bacteriocin type	Primers name	Primers sequence (5'– 3')	cycle	Amplicon size (bp)	Reference
B	colicinB-F<br>colicinB-R	F:AAGAAAATGACGAGAAGACG<br>R:GAAAGACCAAAGGCTATAAGG	95°C – 5min<br>35x (94°C -30s; 60°C-30s; 72°C-1min42s)<br>72°C - 5min	493	(Šmajs et al., 2010)
E1	colicinE1-F<br>colicinE1-R	F:TGTGGCATCGGGCGAGAATA<br>R:CTGCTTCCTGAAAAGCCTTTT	95°C – 5min<br>30x (94°C -30s; 55°C-30s; 72°C - 1min)<br>72°C - 5min	650	
E2	ColE2-F<br>ColE2-R	F:TGATGCTGCTGCAAAAGAG<br>R:TTCAAAGCGTTCCCTACCAC	95°C – 5min<br>25x (94°C -30s; 50°C-20s; 73°C - 1min)<br>72°C - 7min	409	
Ia	ColIa-F<br>ColIa-R	F:GCATGCAAATGACGCTCTTA<br>R:GAGGACGCCAGTTCTCTGTC	95°C – 5min<br>35x (94°C -30s; 55°C-30s; 72°C - 1min)<br>72°C - 7min	473	
Ib	ColIb-F<br>ColIb-R	F:AACGAGTGGGTCGATGATTC<br>R:CCTTTTCTGCGCTCGTATTC	95°C – 5min<br>30x (94°C -30s; 55°C-30s; 72°C - 1min)<br>72°C - 5min	464	
M	ColM-F<br>ColM-R	F:GCTTACCACTTCGCAAAACC<br>R:GAGCGACTCTCCGATAATGC	95°C – 5min<br>35x (94°C -30s; 62°C-30s; 72°C-1min42s)<br>72°C - 5min	429	
Microcin V	microcin V-F<br>microcin V-R	F:CACACACAAAACGGGAGCTGTT<br>R:CTTCCCGCAGCATAGTTCCAT	95°C – 12min<br>30x (94°C -30s; 55°C-30s; 68°C-2min25s)<br>72°C - 3min	680	

Table S2 – Primers used for evaluation of genes related to serum resistance and hemolysis in aEPEC strains

Gene target	Related virulence factor	Primers sequence (5'-3')	cycle	Amplicon size (bp)	reference
ehxA	Enterohemolysin	F: GGTGCAGCAGAAAAAGTTGTAG<br>R: TCTCGCCTGATAGTGTTTGGTA	94ºC – 5 min,<br>30x (94ºC – 30 s; 57ºC – 1 min; 72ºC – 90 s)<br>72ºC – 7 min	1551	(Schmidt et al., 1995)
hlyA	alpha-hemolysin	F: AACAAGGATAAGCACTGTTCTGGCT<br>R: ACCATATAAGCGGTCATTCCCGTCA	94ºC – 5 min,<br>30x (94ºC – 1 min; 63ºC – 30 s; 72ºC – 3 min)<br>72ºC – 10 min	1177	(Yamamoto et al., 1995)
iss	increasing serum survival	F: TCACATAGGATTCTGCCG<br>R: AGAAATCAAAAGGTGGCC	95ºC – 12 min,<br>25x (94ºC - 30 s; 63ºC - 30 s; 68ºC - 3 min)<br>72ºC – 10 min	607 pb	(Dezfulian et al., 2003)
traT	Outer membrane protein associated with inhibition of the classical pathway of complement activity and serum survival	F: GGTGTGGTGCGATGAGCACAG<br>R: CACGGTTCAGCCATCCCTGAG	95ºC – 12 min,<br>25x (94ºC - 30 s; 63ºC - 30 s; 68ºC - 3 min)<br>72ºC – 10 min	290 pb	(Johnson & Stell, 2000)

Table S3 - Multiplex used for classification of ExPEC intrinsic virulence and uropathogenic potential

Target genes	Related VFs	Primers sequence (5'– 3')	Cycle	Amplicon size (bp)	Reference
iutA	Aerobactin	F: GGCTGGACATCATGGGAACTGG<br>R: CGTCGGGAACGGGTAGAATCG	95°C–12min<br>25x (94°C- 30s; 63°C-30s; 68°C-3min)<br>72°C - 10min	300	(Johnson et al., 1997)
kpsMT II	Group 2 capsule	F: GCGCATTTGCTGATACTGTTG<br>R: CATCCAGACGATAAGCATGAGCA		272	(Johnson & Stell, 2000)
papC	P fimbriae	F: GACGGCTGTACTGCAGGGTGTGGCG<br>R: ATATCCTTTCTGCAGGGATGCAATA	95°C–5min<br>25x (94°C- 2min; 65°C-1min; 72°C-2min)<br>72°C - 5min	328	(Le Bouguenec et al., 1992)
sfaDE	S fimbriae	F: CGGAGGAGTAATTACAAACCTGGCA<br>R: CTCCGGAGAACTGGGTGCATCTTAC		410	
*afaBC*III	Afimbrial adhesin-3	F: GCTGGGCAGCAAACTGATAACTCTC<br>R: CATCAAGCTGTTTGTTCGTCCGCCG		750	
vat	Vacuolating autotransporter toxin	F: TCAGGACACGTTCAGGCATTCAGT<br>R: GGCCAGAACATTTGCTCCCTTGTT	94 °C – 15 min<br>30x (94 °C – 30s; 63 °C – 90s; 72 °C -90s)<br>72 °C – 10min	1100	(Spurbeck et al., 2012)
fyuA	Yersiniabactin	F: GTAAACAATCTTCCCGCTCGGCAT<br>R: TGACGATTAACGAACCGGAAGGGA		850	
yfcV	Yfc fimbriae	F: ACATGGAGACCACGTTCACC<br>R: GTAATCTGGAATGTGGTCAGG		292	

Table S4 - Multiplex primers and conditions used to search for ExPEC pathogenicity islands[a]

	PAI target	Primer name	Primers sequence (5'– 3')	Amplicon size (bp)	Virulence factors present in the PAI[b]	Cycle
**Multiplex A**	II_{536}	orf1up<br>orf1down	F: CATGTCCAAAGCTCGAGCC<br>R: CTACGTCAGGCTGGCTTTG	1000	α-hemolysin and P fimbriae	94°C–5min<br>30x (94°C- 1min; 58°C-1min; 72°C-1min)<br>72°C - 7min
	III_{536}	sfaAI.1<br>sfaAI.2	F: CGGGCATGCATCAATTATCTTTG<br>R: TGTGTAGATGCAGTCACTCCG	200	S fimbriae, vacuolating autotransporter toxin, and Salmochelin	
	IV_{536}	IRP2 FP<br>IRP2 RP	F: AAGGATTCGCTGTTACCGGAC<br>R: TCGTCGGGCAGCGTTTCTTCT	300	Yersiniabactin	
	II_{CFT073}	cft073.2Ent1<br>ecft073.2Ent2	F: ATGGATGTTGTATCGCGC<br>R: ACGAGCATGTGGATCTGC	400	P fimbriae and iron responsive element (Ire)	
**Multiplex B**	I_{536}	I.9<br>I.10	F: TAATGCCGGAGATTCATTGTC<br>R: AGGATTTGTCTCAGGGCTTT	1800	α-hemolysin, CS12 fimbriae, and F17-like fimbriae	94°C–5min<br>30x (94°C- 1min; 55°C-1min; 72°C-1min)<br>72°C - 10min
	I_{J96}	papGIf<br>papGIr	F: TCGTGCTCAGGTCCGGAATTT<br>R: TGGCATCCCACATTATCG	400	α-hemolysin and P fimbriae	
	II_{J96}	hlyD<br>cnf	F: GGATCCATGAAAACATGGTTAATGGG<br>R: GATATTTTTGTTGCCATTGGTTACC	2300	α-hemolysin, Prs fimbriae, and CNF1 (Cytotoxic necrotizing factor 1)	
	I_{CFT073}	RPAi<br>RPAf	F: GGACATCCTGTTACAGCGCGCA<br>R: TCGCCACCAATCACAGCGAAC	930	α-hemolysin, P fimbriae, Aerobactin, capsule group II, secreted autotransporter toxin	

[a] Primers described by Sabaté et al. (2006) were used in an optimized PCR multiplex as described by da Silva et al., (2017).

[b] Known virulence factors present in the PAIs evaluated.

Table S6 – Phylogenetic distribution of the aEPEC strains evaluated [n (%)]

Phylogroup	Control[a]	Case[b]	TOTAL
**A**	12 (36.3)	20 (25.6)	32 (28.8)
**B1**	14 (42.4)	40 (51.2)	54 (48.6)
**B2**	4 (12.1)	11 (14.1)	15 (13.5)
**C**	0 (0)	0 (0)	0 (0)
**D**	0 (0)	1 (1.3)	1 (0.9)
**E**	3 (9)	6 (7.7)	9 (8.1)
**F**	0 (0)	0 (0)	0 (0)
**TOTAL**	33	78	111

[a] aEPEC strains isolated from the stools of healthy individuals

[b] aEPEC strains isolated from diarrhea

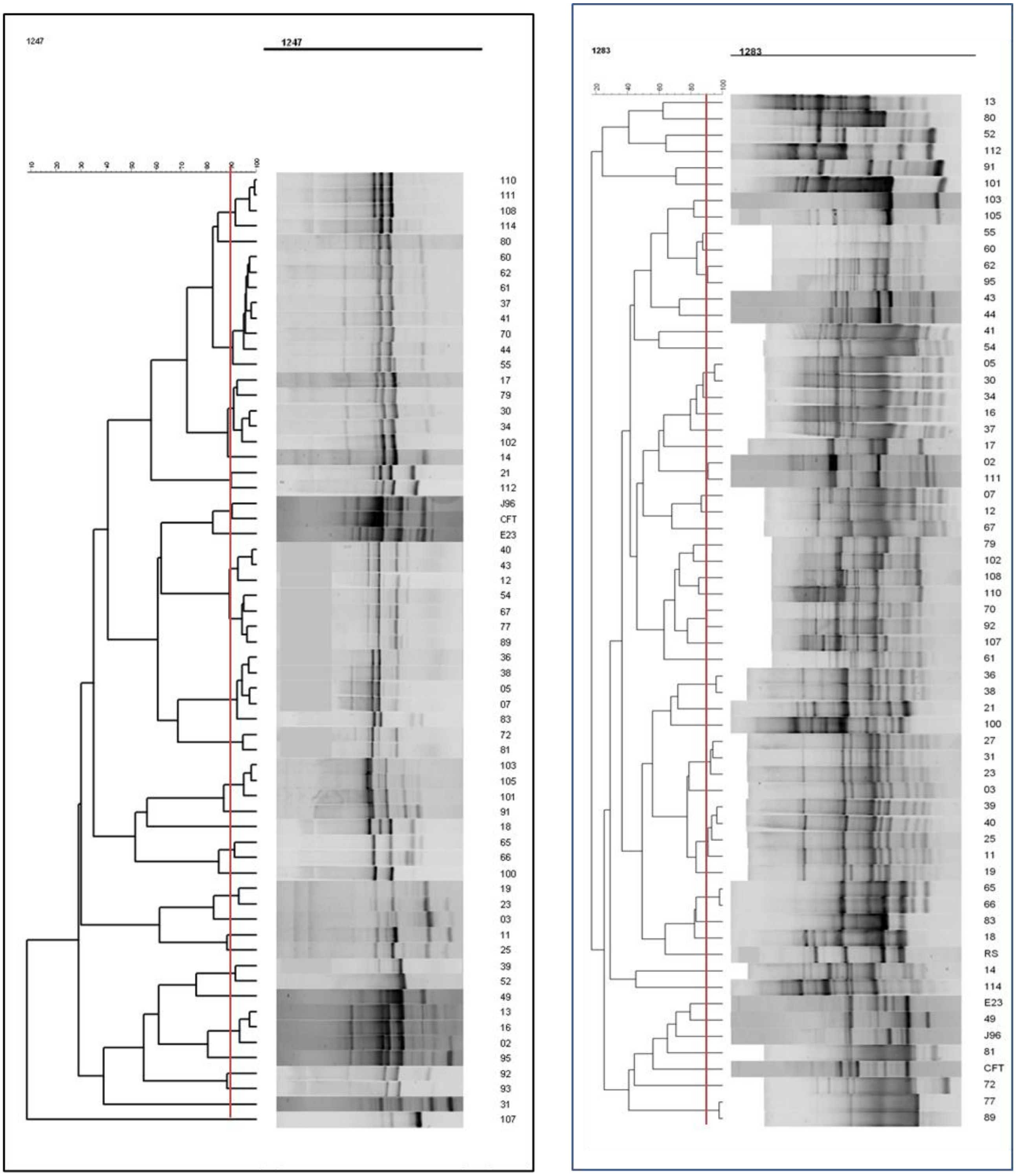

Figure S1 – Clonal evaluation of aEPEC strains using RAPD amplification patterns. Primers 1247 (A) and 1283 (B) were used to evaluate clonal relationship of aEPEC strains. Red line showed the cutoff point of 90% of amplification identity. The amplification patterns were used together to determine the strains clonality. Strains that displayed 90% of identity in both PCRs are considered to belong to the same phylogenetic group.

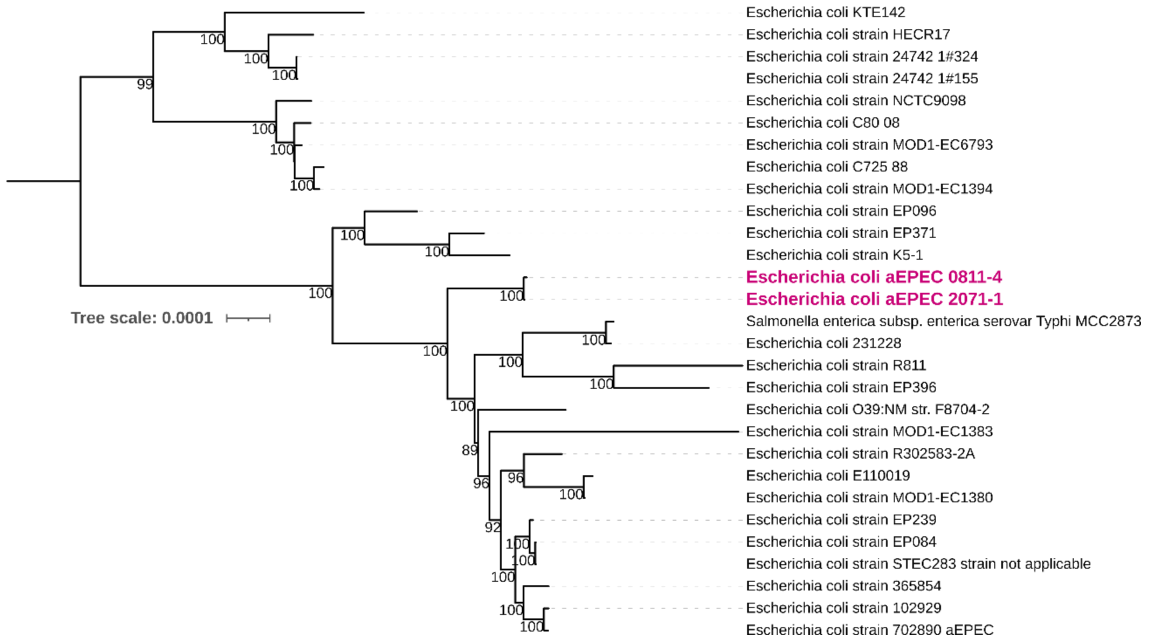

Figure S2 – Clonal relationship between aEPEC 0811-4 and 2071-1. The phylogenetic tree was built using 27 similar genomes identified by the MASH-MinHash tool. The identified hybrid aEPEC/ExPEC strains were highlighted in bold and pink. Bootstrap ≥ 85 was displayed in tree nodes.

References

da Silva, L. C., de Mello Santos, A. C., & Silva, R. M. (2017). Uropathogenic *Escherichia coli* pathogenicity islands and other ExPEC virulence genes may contribute to the genome variability of enteroinvasive *E. coli*. *BMC Microbiology, 17*(1), 68. https://doi.org/10.1186/s12866-017-0979-5

Dezfulian, H., Batisson, I., Fairbrother, J. M., Lau, P. C. K., Nassar, A., Szatmari, G., & Harel, J. (2003). Presence and Characterization of Extraintestinal Pathogenic *Escherichia coli* Virulence Genes in F165-Positive *E. coli* Strains Isolated from Diseased Calves and Pigs. *Journal of Clinical Microbiology, 41*(4), 1375. https://doi.org/10.1128/JCM.41.4.1375-1385.2003

Johnson, J. R., Stapleton, A. E., Russo, T. A., Scheutz, F., Brown, J. J., & Maslow, J. N. (1997). Characteristics and prevalence within serogroup O4 of a J96-like clonal group of uropathogenic *Escherichia coli* O4:H5 containing the class I and class III alleles of papG. *Infection and Immunity, 65*(6), 2153–2159. https://doi.org/10.1128/IAI.65.6.2153-2159.1997

Johnson, J. R., & Stell, A. L. (2000). Extended virulence genotypes of *Escherichia coli* strains from patients with urosepsis in relation to phylogeny and host compromise. *Journal of Infectious Diseases, 181*(1), 261–272. https://doi.org/10.1086/315217

Le Bouguenec, C., Archambaud, M., & Labigne, A. (1992). Rapid and specific detection of the *pap*, *afa*, and *sfa* adhesin-encoding operons in uropathogenic *Escherichia coli* strains by polymerase chain reaction. *Journal of Clinical Microbiology, 30*(5), 1189–1193. https://doi.org/10.1128/JCM.30.5.1189-1193.1992

Sabaté, M., Moreno, E., Pérez, T., Andreu, A., & Prats, G. (2006). Pathogenicity island markers in commensal and uropathogenic *Escherichia coli* isolates. *Clinical Microbiology and Infection, 12*(9), 880–886. https://doi.org/10.1111/j.1469-0691.2006.01461.x

Schmidt, H., Beutin, L., & Karch, H. (1995). Molecular analysis of the plasmid-encoded hemolysin of *Escherichia coli* O157:H7 strain EDL 933. *Infection and Immunity, 63*(3), 1055–1061. https://doi.org/10.1128/IAI.63.3.1055-1061.1995

Šmajs, D., Micenková, L., Šmarda, J., Vrba, M., Ševčíková, A., Vališová, Z., & Woznicová, V. (2010). Bacteriocin synthesis in uropathogenic and commensal *Escherichia coli*: colicin E1 is a potential virulence factor. *BMC Microbiology, 10*(1), 288. https://doi.org/10.1186/1471-2180-10-288

Spurbeck, R. R., Dinh, P. C., Walk, S. T., Stapleton, A. E., Hooton, T. M., Nolan, L. K., Kim, K. S., Johnson, J. R., & Mobley, H. L. T. (2012). *Escherichia coli* Isolates That Carry *vat* , *fyuA* , *chuA* , and *ycfV* Efficiently Colonize the Urinary Tract. *Infection and Immunity*, *80*(12), 4115–4122. https://doi.org/10.1128/IAI.00752-12

Yamamoto, S., Terai, A., Yuri, K., Kurazono, H., Takeda, Y., & Yoshida, O. (1995). Detection of urovirulence factors in *Escherichia coli* by multiplex polymerase chain reaction. *FEMS Immunology and Medical Microbiology, 12*(2), 85–90. https://doi.org/10.1016/0928-8244(95)00053-A
